# Consequences of *in utero* exposure to Zika virus in offspring of AG129 mice

**DOI:** 10.1038/s41598-018-27611-x

**Published:** 2018-06-20

**Authors:** Justin G. Julander, Venkatraman Siddharthan, Albert H. Park, Elizabeth Preston, Pranav Mathur, Michael Bertolio, Hong Wang, Katherine Zukor, Arnaud J. Van Wettere, Donal G. Sinex, John D. Morrey

**Affiliations:** 1 Institute for Antiviral Research, Animal, Dairy, and Veterinary Sciences Department, 5600 Old Main Hill, Utah State University, Logan, Utah 84322-5600 USA; 2 Division of Otolaryngology- Head and Neck Surgery, University of Utah, 50 N Medical Dr., Salt Lake City, UT USA; 3 Audiology Division, Department of Communicative Disorders and Deaf Education, Utah State University, 2620 Old Main Hill, Logan, UT 84322-2620 USA; 4 Department of Neurobiology and Anatomy, University of Utah, 1795 E South Campus Drive, Salt Lake City, UT 84112 USA; 5 Utah Veterinary Diagnostics Laboratory, Utah State University, 950 E 1400 N, Logan, UT 84341 USA; 6 Department of Biology, Utah State University, 5305 Old Main Hill, Logan, UT 84322-5305 USA

## Abstract

Zika virus (ZIKV) can cause various diseases in offspring after congenital infection. The purpose of this study was to identify disease phenotypes in pups exposed to ZIKV *in utero*. Female interferon-α/β, -γ receptor knockout mice (AG129) were infected intraperitoneally with ZIKV 7.5 days’ post coitus (dpc). Viral RNA, antigen and infectious virus were detected in some, but not all, maternal and fetal tissues at various times during gestation. Fetuses of infected dams had significant intrauterine growth restriction (IUGR), which was more pronounced as females neared parturition. Pups born to infected dams were significantly smaller and had significantly shortened skull lengths, as determined by measurement with a caliper and by micro-CT analysis, as compared with age-matched controls. Growth rates of exposed pups after birth, however, was similar to sham-exposed offspring. Viral RNA was detected in pups of infected dams after birth. A lower survival rate was observed in neonates exposed to ZIKV *in utero*. A mortality rate of over 50%, attributed to consequences of ZIKV infection, occurred after birth in pups born to infected dams. A transient hearing loss was observed in some animals exposed to virus *in utero*. No motor deficits or cognitive deficits were detected using running wheel or viral paresis scoring assays. Abnormalities in offspring included smaller size, shorter skull length and increased neonatal mortality, while the only functional deficit we could detect was a low incidence of transient hearing loss.

## Introduction

Zika virus (ZIKV) infection typically results in a mild, self-limiting infection that may include rash, fever, muscle aches, joint pain, headache, and other manifestations such as Guillain-Barre syndrome^1^. In addition to the typical infection routes, ZIKV can also be acquired through congenital exposure from a woman to her developing fetus^2^. Infection during pregnancy can result in severe disease in exposed fetuses. One of the most debilitating consequences of fetal infection is microcephaly^3^. Microcephaly, however, does not occur in all cases of fetal ZIKV infection. A broad spectrum of disease has been reported in neonates born to mothers that were infected during pregnancy, which include low birth weight, arthrogryposis, hearing deficiencies, ocular disease, ventriculomegaly and cerebellar hypoplasia^4,5^. Neonatal death as a consequence of congenital Zika virus infection has been reported between 5.1–27.3%^4,6^. Although the effects of intrauterine ZIKV infection may be dramatic, asymptomatic neonates are also reported in women exposed to the virus during pregnancy^7^.

Modeling ZIKV in mice is difficult, since wild-type strains of mice are typically resilient to infection^8^. For this reason, various immunocompromised mouse strains have been used to model ZIKV infection. Since the interferon pathway is key for controlling many flavivirus infections in rodents, various strains lacking key elements of the interferon (IFN) pathway have been used to model various aspects of disease. Adult interferon receptor deficient strains (IFNAGR^−/−^, IFNAR^−/−^), IFNAR Ab functional knockouts, and STAT1 or STAT2 knockout rodents infected with ZIKV allow replication of the virus and manifestation of various aspects of disease^9–14^. Researchers have also developed a model of congenital ZIKV disease in which wild-type males were mated with IFNAR^−/−^ females, resulting in susceptible IFNAR^+/−^ fetuses that display gestational age-dependent effects of ZIKV infection on offspring development^15,16^. The presence of IFN responsiveness in heterozygote fetuses was later shown to be involved in pathogenesis^17^. While these models may lack certain components of the IFN response pathway, immunocompromised mouse models have utility in understanding congenital infection with ZIKV^18,19^. Neonatal mice, which lack a fully developed immune response, may also be productively infected^20–22^.

Several mouse models of congenital ZIKV infection have been reported, but these typically focus on the fetus^16,23^ or just after birth^24,25^. We have described some outcomes of *in utero* ZIKV exposure in a hamster model^26^, primarily cannibalization of offspring, which is common in rodents that are diseased or otherwise stressed. In the present study, we investigated the consequences of ZIKV infection on the offspring of AG129 mice that were infected on 7.5 dpc of pregnancy. Various aspects of human congenital Zika were demonstrated in newborns, including smaller size and reduced skull size. Long term effects included an increased mortality rate and transient hearing loss in pups exposed *in utero* to ZIKV. Activity wheel and viral paresis score assays used to assess cognitive and motor function did not demonstrate any long-term deficits. This model of congenital ZIKV infection will allow for further studies to better understand the consequences of intrauterine exposure and can provide a tool to identify methods of control.

## Materials and Methods

### Virus

A Malaysian isolate of ZIKV (P 6-740, Robert Tesh, WRCEVA, Galveston, TX) was passaged 2 times in Vero 76 cells. Infected cells were frozen once, thawed, centrifuged to remove cell debris, and aliquoted in frozen stocks. The frozen stock had a titer of 10^5.6^ 50% cell culture infectious doses (CCID_50_/mL).

### Mice

AG129 mice^27^ were produced from in-house colonies at Utah State University. Timing of breeding was determined in mice by identifying vaginal plugs, which signified 0.5-days post-coitus (dpc). At 7.5 dpc, pregnant mice were injected subcutaneously (s.c.) in the inguinal area with 100 CCID_50_ of ZIKV. In two of seven studies, estrous was stimulated with an i.p. injection of 1 IU of pregnant mare serum gonadotropin (PMSG) followed two days later with an injection of 1 IU of human chorionic gonadotropin (hCG).

Necropsies were performed on 3, 5, 7, 9, 10, 12 and 14-days post-virus inoculation (dpi), corresponding with 10.5, 12.5, 14.5, 16.5, 17.5 and 18.5 dpc, or post-natal day (PND) 0 (day of birth), respectively. Two separate studies were conducted for the first and second half of the time-course above. Two to 3 pregnant mice were sacrificed at each time-point. Upon necropsy, a maternal liver, spleen and/or brain sample was collected to determine peripheral titer in the female. The uterine horns, containing fetuses, were removed and washed in 95% ethanol. The uterus was aseptically opened to extract individual conceptuses, including the placenta. The complete fetal unit was washed in 95% ethanol followed by rinsing in phosphate buffered saline. The placental disk was peeled from the rest of the conceptus and then the fetus was removed from the embryonic sac, washed again with ethanol and PBS, and both placenta and fetus were measured for crown-rump length (CRL), occipito-frontal diameter (OF), head length (HL) and weight, followed by processing for virus titration by PCR. At later times of necropsy (14.5 and 16.5 dpc), the heads and viscera of the fetuses were processed separately or only head virus titers were assessed. Three to 5 concepti from each female were prepared for RT-PCR and infectious viral assays. Tissue samples were placed in plastic bags containing ~3:1 volume:weight of minimal essential medium and homogenized. The remaining tissues were fixed in freshly prepared 4% paraformaldehyde and processed for immunohistochemistry (IHC).

Pups born to infected and sham-infected dams were measured at various times after birth, including CRL, OF, HL and weight and some pups were necropsied to determine the presence of virus in tissues. Morbidity and mortality were also monitored after birth for a total of 69 and 64 pups from ZIKV-exposed and sham-exposed pups, respectively. Since females typically succumbed to viral illness within a week of parturition, age- and gestation-matched controls were included in each study to serve as surrogates for raising pups born to infected dams. Functional assays (running wheel assay, paresis scoring and hearing tests) were used to assess the consequences of intrauterine ZIKV exposure.

### RT-PCR

A volume of 0.1 mL of tissue homogenate was added to 900 µL Trizol Reagent™ and RNA was extracted per manufacturer’s instructions and suspended in nuclease-free water. For amplification reactions, 5 μL of master mix containing polymerase, primers and Rapid Probe, labeled with a FAM fluorophore and TAMRA quencher, was mixed with 5 μL of RNA. ZIKV RNA was first reverse transcribed for 10 minutes at 55 °C, heated to 95 °C for 20 seconds. The PCR was 40 cycles at 95 °C for 1 second, and 55 °C for 20 seconds. A standard curve was generated with known copy-number synthetic RNA, which was used to quantify samples of unknown concentration. Primers for GAPDH were used to quantify total RNA levels and to adjust the relative ZIKV RNA levels.

### Infectious assay

An infectious culture assay was used as previously described^26^. Briefly, a volume of 20 µL homogenized tissue was mixed with 180 µL of MEM, serially diluted, added to a cell-culture dish (96-well plate) seeded with Vero cells and incubated in a CO_2_ incubator at 37 °C for 5 days. At the end of the incubation cells were fixed and immunocytochemistry was performed against anti-flavivirus group antigen (EMD Millipore, CA).

### Histopathology and Immunohistochemistry

Two to 4 pups from each litter were prepared for histopathology and immunohistochemistry (IHC) as previously described^26^. Briefly, tissues were fixed in 4% paraformaldehyde followed by transfer 24 h later to 70% ethanol. Tissues were trimmed, paraffin-embedded, and sectioned (5–8 µm) and stained with hematoxylin and eosin (H&E). Stained slides with H&E were analyzed for histologic changes by a board-certified veterinary pathologist. The identity of the slides was blined to the pathologist. For immunohistochemistry, tissue sections were blocked and then incubated overnight with ZIKV-specific antibody (Ab), followed by incubation with a labeled secondary Ab as previously described^26^. Images were captured using Zeiss microscope, AxioVision 4.0.1 and processed using Adobe Photoshop (CC 2018).

To perform a more thorough survey of select fetal brains for ZIKV, immunohistochemistry was performed on serial sections (1 for every 50 coronal sections) of 3 fetuses from dam #15 and 4 fetuses from dam #8 (both necropsied on 9 dpi, 16.5 dpc, Table 1). Analysis included fetuses that were either positive or negative for ZIKV immunoreactivity (IR) and also included one fetus that was negative for ZIKV IR, but was positive for infectious virus (Table 1).

**Table 1:** Viral and histological analysis of tissues collected at different time-points from pregnant mice infected with ZIKV on 7.5 dpc.

dpc (dpi)	Inoculation	Dam ID	ZIKV RT-PCR		Infectious virus		ZIKV IR^a^			Histopath. and morphologic^b^			Total
			Placenta pos./total	Fetus^c^ pos./total	Placenta pos./total	Fetus pos./total	Uterus^d^ pos./total	Placenta pos./total	Fetus pos./total	Uterus^d^ pos./total	Placenta pos./total	Fetus pos./total	
10 (3)	Sham	26	0/2	0/2	0/2	0/2	0/4	0/4	0/4	0/4	1/4	0/4	6
10 (3)	ZIKV	9	1/2	1/2	—	—	—	—	—	—	—	—	—
12 (5)	ZIKV	4	1/1	1/1	2/2	1/2	2/2	0/2	0/2	0/2	0/2	0/2	4
12 (5)	ZIKV	13	3/3	3/3	—	—	1/2	0/2	0/2	0/2	0/2	0/2	2
12 (5)	ZIKV	18	3/3	2/3	0/1	0/1	1/2	0/2	0/2	0/2	0/2	0/2	3
14 (7)	ZIKV	2	3/3	2/4	3/3	0/3	3/4	0/4	0/4	3/4^e^	2/4^f^	0/4	7
14 (7)	ZIKV	11	1/2	3/3	0/2	0/2	2/3	0/3	0/3	0/3	3/3^f^	0/3	5
14 (7)	ZIKV	19	—	3/3	3/3	1/3	3/4	3/4	0/4	4/4^e^	4/4^f^	0/4	7
16 (9)	ZIKV	15	3/3	0/3	1/1	0/1	2/3	1/3	2/3	2/3^e^	2/3^f^	2/3^g^	4
16 (9)	ZIKV	8	3/3	3/3	4/4	1/4	2/4	2/4	0/4	2/4^e^	1/4^f^	0/4	8
16 (9)	ZIKV	28	1/1	1/1	—	—	—	—	—	—	—	—	—

^a^Zika virus immunoreactivity as determined by immunohistochemistry.

^b^Histopathological analysis performed on fixed and H&E-stained tissue sections.

^c^Fetal tissue analyzed included complete fetus or fetal brain.

^d^A section of uterus in contact with the conceptus.

^e^Metritis, characterized by multifocal infiltration of the myometrium interstitium by a small to moderate number of mononuclear cells and few neutrophils.

^f^Placentitis, characterized by a thin band of karyorrhectic debris and eosinophilic fluid admixed with few presumptive degenerated neutrophils at the interface between the decidua basalis and the spongiotrophoblast.

^g^Meningitis characterized by multifocal infiltration of few neutrophils within the leptomeninges.

^—^, Not available; dpi, days post-viral injection; dpc, days post-coitus.

Cochleae from acutely infected adult AG129 mice (10–29 dpi) were quickly removed from temporal bone and decalcified within 10% EDTA at 4 °C. This was followed by dehydration through 10 sucrose for 30 minutes at room temperature, then kept at 4 °C in 15% sucrose overnight. Tissues were then embedded in OCT within cryomould with the round window facing down. Cryosections were cut at 20 µm and mounted on a glass slide pretreated with poly-l-lysine. For IHC staining, sections were rinsed with PBS, blocked with 10% donkey serum and 0.5% Triton X-100 in PBS for 1 hour. Primary antibodies (neuronal marker, NeuN and ZIKV) were added and incubated overnight at 4^0^C. Secondary antibodies conjugated to Alexa-488 and 568 were added for 2 hours at room temperature. After rinsing in PBS, a coverslip was added to sections with Vectshield mounting medium with DAPI (H-1200, Vecotor Laboratories). Fluorescent images were obtained using 10X and 20X objective lenses with a laser scanning confocal microscope (Zeiss, LSM710).

### MicroCT

Images were acquired using an Inveon trimodality PET/SPECT/CT scanner (Siemens Preclinical Solutions, Knoxville, TN). CT images consisting of 360 degrees and 720 projections were acquired first with the axial field of view of 60.28 mm and transaxial field of view of 30.89 mm. The large field of view allowed two specimens to be scanned at a time. The exposure time was 2,200 msec with a detector setting at 80 kVp and 150 µA. Data were reconstructed onto a 1312 × 1312 × 2560 image matrix using the Siemens Inveon FeldKamp software package. The effective image pixel size was 23.55 µm. Crown-rump length, skull length, cranial height and bi-parietal diameter were obtained.

### Hearing assessment

Hearing was assessed in congenitally exposed mice using auditory brainstem response (ABR) with the use of the Biologic NavPro System. Animals were sedated with ketamine/xylazine and needle electrodes were placed behind the test ear as the reference electrode (A1/A2), on top of the head as the active electrode (Cz) and on the back just anterior to the tail (ground) of the mice. An insert was placed in the ear to deliver the stimulus, which was a tone burst at 4000, 6000, or 8000 Hz. Blackman ramping was used with an alternating polarity and a rate of 21.70 ms. The low filter was 50 Hz and the high filter was 1500 Hz with artifact rejection at 23.80. The mice were placed in a custom-designed, 16-cubic foot copper faraday cage (BioQuip Products) constructed with sound-proofed home-theater drop-ceiling panels and pyramidal sound-deadening foam (Home Theater Supplies), which was grounded to control for external electrical noise. To determine the threshold, responses were collected starting at 85–95 dB and decreasing until no responses were obtained. Responses at threshold and no response were repeated to verify the accuracy of the response.

An in-house designed ABR instrument was also used for hearing assessment, which utilized LabView 8.2 (National Instruments), an NI USB-6212 data acquisition board connected to a Pyle Pro PTA2 power amp driver and an acoustic pre-amp/attenuator (Marchand Electronics) connected through a SCB-68 shielded connector block (National Instruments) to generate digital acoustic signals. The system outputs 0.025 millisecond clicks (40 kHz) or pure tone frequencies from 8 to 32 kHz pulsing at an 11 Hz frequency. Electrodes were placed as described above. A single DT-48 multi-field hi-fidelity magnetic speaker (Beyerdynamic Inc.) attached to a polyethylene tube, which was placed in the ear to deliver sound to the ear canal. A series of responses generated using a stimulus of 8 kHz pure tone pips were collected with 5–10 dB incremental steps from 25 to 100 dB SPL. The response at each dB level was collected twice for confirmation. The signal was amplified 10X with a Dagan head-stage pre-amp and filtered from 100 Hz to 1,000 Hz using a Dagan EX-4-400 differential amplifier. A minimum of 500–1,000 evoked responses were averaged and digitized.

Terminal cochelar action potentials (CAP) were collected on animals with hearing deficits. Mice were anesthetized with ketamine/xylazine (40, 5 mg/kg) and were placed on a feedback-controlled heating pad to keep the body temperature at 37.5 °C. The bulla of the middle ear was exposed and a small window was made using miniature scalpel without damaging the tympanic membrane. A monopolar needle electrode (EL452, Biopac Systems) was placed on both the cochlear promontory and round window membrane using a surgical microscope. A reference electrode was placed subcutaneously on the opposite side of the head. CAP was measured after exposure to an acoustic click stimulus with a range of 10 to 100 dB. A minimum of 100 measurements was averaged.

### Confocal microscopy of hair cells

Cochlear samples, collected from four mice from two separate studies, were evaluated for hair cell loss. The skulls of the animals were fixed, after removal of the brain at necropsy, in freshly prepared 4% paraformaldehyde at 4 °C. Fixed cochleas were dissected and stained for actin using Phalloidin (Alexa Flour^TM^ 488, Cat# A12379), diluted 1:500 in PBS and incubated overnight at 4 °C. Samples were then mounted on slides and imaged on confocal laser scanning microscope (Model FV1000, Olympus, Tokyo, Japan). Outer hair cell (OHC) loss from cochleae imaged was counted manually from all the regions of Apex, mid and basal turn and calculated as % OHC loss.

### Running wheel test

Mice exposed in utero to ZIKV were placed for 7 days on a Techniplast Activity Cage Systems running wheel that counts the number of rotations in either direction due to mouse activity. Data were compiled using the Vital View Activity Software 4.1 (Starr Life Sciences Corp). Age-matched mice that were not exposed to ZIKV were run in parallel to serve as naïve controls. Activity graphs were also generated to determine the effect of ZIKV exposure on circadian rhythm.

### Viral paresis scale (VPS)

Mice were analyzed for signs of tail and hind-limb paresis/paralysis using a sensitive, open-field assay modified from the Basso Mouse Scale used to assess paralysis in spinal cord injured mice^28^ and a test used to track paralysis in amyotrophic lateral sclerosis mouse models^29^. Each mouse was placed on a tabletop and allowed to roam freely for about 4 minutes (min). Hind-limb function was scored on a 7-point scale detailed in Suppl. Table 1 by researchers who were blind to the infection status of each group. Scoring was based on 4 main categories: tail position during walking, miss-step severity, weight bearing and joint movement. Miss-step severity was scored only on assessable walking passes, which was defined as a pass in which the animal moved 3 body lengths at a consistent speed and without turning^28^. Separate scores were given for the left and right hind-limbs to assess if symptoms were bilateral or unilateral.

### Ethics statement

This study, including veterinary care and experimental procedures, was conducted in accordance with the approval of the Institutional Animal Care and Use Committee of Utah State University (USU) under the approved protocol #2550. The work was performed in the AAALAC-accredited Laboratory Animal Research Center at Utah State University.

### Statistical analysis

All statistical analyses, including one- and two-way ANOVA, student’s t-test, and Wilcoxon log-rank test were performed using Prism 7 (GraphPad Software, Inc). The analysis used is noted in the figure legend.

### Data availability statement

Raw data files will be made freely available upon request.

### Author Summary

Congenital exposure to ZIKV results in fetal and placental infection and contributes to microcephaly and other serious disease. Mouse neonates born to infected dams had reduced body size and skull lengths indicative of intrauterine growth restriction. Infectious virus and ZIKV RNA was detected in some, but not all, fetal and neonatal mice. A low incidence of hearing loss was observed, but no motor or cognitive deficits were observed.

## Results

### Time-course of viral infection of congenitally exposed mice

**Figure 1:**
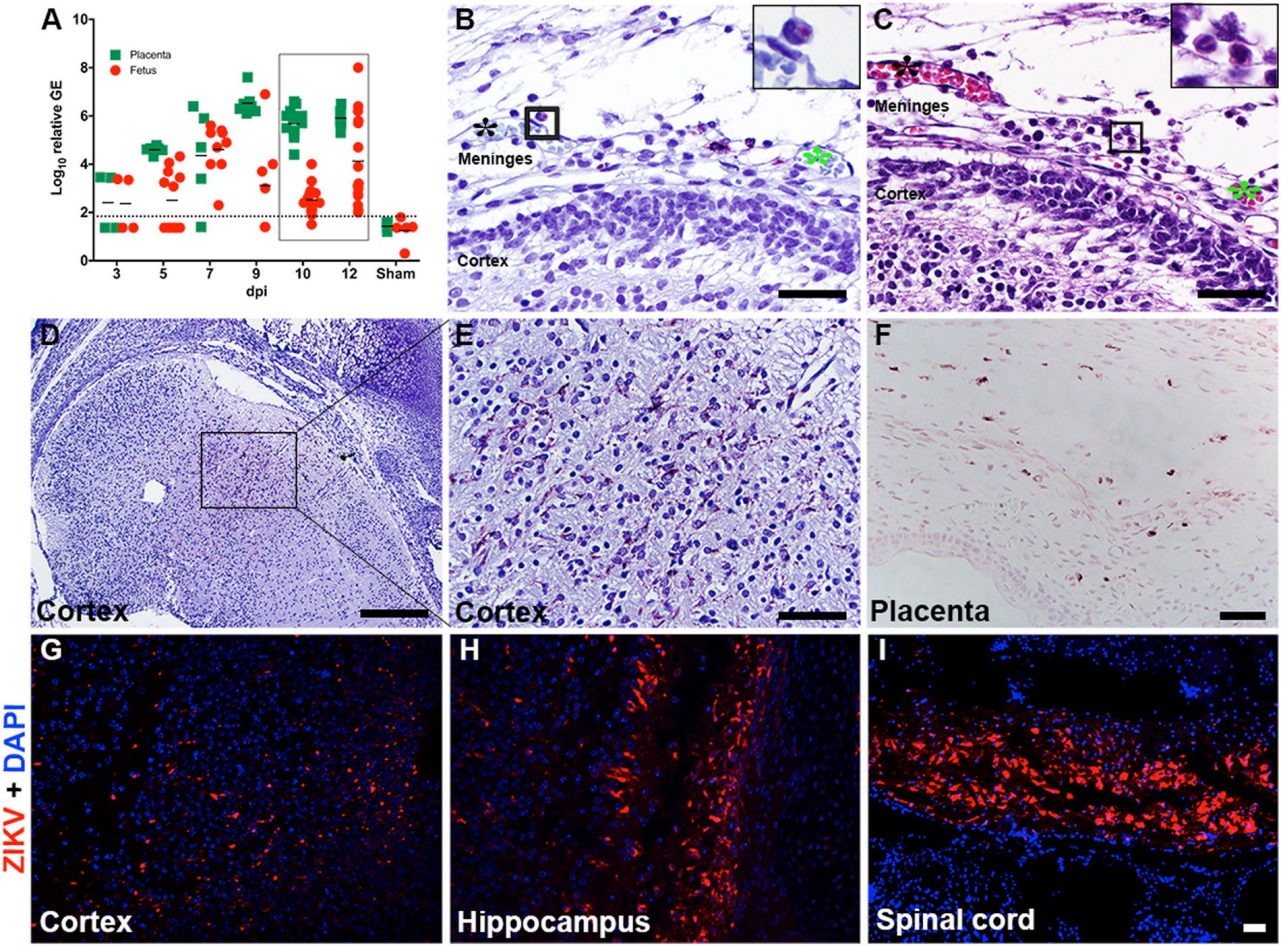
ZIKV titers and histopathology in maternal and fetal tissues of AG129 mice challenged 7.5 dpc with ZIKV. (**A**) Time-course of ZIKV RNA levels in placentas, fetuses, and maternal liver and brain. These are representative data from two separate studies (delineated by the black box). Sham tissues were collected on 5 dpi and the dashed line indicates limit of assay sensitivity. Representative sections of various tissues immunoreactive for ZIKV using peroxidase immunostaining, including (**B**,**D**,**E**) fetal cortex and (**F**) placenta. Panels B and C are sequential sections (landmarks marked with *) showing (**B**) the presence of ZIKV IR in infiltrating neutrophils (boxed insert) and (**C**) hematoxylin and eosin stained section, which shows infiltrating band neutrophil with U-shaped nucleus. (**B**,**C** boxed insert, arrowhead). (**G**) ZIKV IR in sections of the fetal cortex was confirmed using immunofluorescent staining. ZIKV IR was also demonstrated by immunofluorescent staining in sections of fetal (**H**) hippocampus and (**I**) spinal cord.

A time-course study was conducted to determine the course of viral infection in fetuses at various times between 3 and 12 days’ post-virus inoculation (dpi), which coincides with early gestation around the time the placenta is forming until just before parturition. Virus was initially detected in the placenta and fetal tissue samples on 3 dpi (10.5 dpc) by RT-PCR, although viral RNA was not observed in all samples tested (Fig. 1A, Table 1). Titers increased by 3 log_10_ at 9 dpi (16.5 dpc) in placentas and consistent detection of ZIKV RNA in placental samples was observed to parturition (Fig. 1A). Viral RNA titers in fetuses were also observed to increase over time, with the majority of fetuses having detectable ZIKV RNA beginning on 7 dpi (14.5 dpc) (Fig. 1A). However, some fetuses from each time-point had low or undetectable ZIKV RNA titers, demonstrating the variability of congenital infection in AG129 mice when infected with this particular strain and dose. The majority of fetuses had detectable viral RNA titers just prior to parturition, but a wide range of viral RNA titers was observed (Fig. 1A). No viral RNA was detected in fetal, placental, or maternal tissues from sham-infected mice collected 3 dpi (Table 1) and 5 dpi (Fig. 1A).

Fetuses, placentas and uterus from infected or sham-infected dams were further evaluated for the presence of infectious virus, viral antigen and histopathological changes to characterize congenital infection in this model. Infectious virus was frequently observed in placental homogenates co-cultivated on Vero cells (Table 1). In fetal tissue homogenates, however, infectious ZIKV was less frequently detected (Table 1).

Although viral RNA titers, ZIKV immunoreactivity (IR) and infectious virus were detected in fetal and maternal tissues, no or minimal histologic lesions were observed in fixed tissues taken at various times after virus challenge except for in the placenta. One consistent observation in placental samples from infected females on 7 and 9 dpi (Table 1) was a thin band of karyorrhectic debris and eosinophilic fluid admixed with a few presumptive degenerated neutrophils at the interface between the decidua basalis and spongiotrophoblast layers, that was interpreted as placentitis. Histopathologic evaluation of fetal brain sections demonstrated minimal neutrophilic infiltration in the meninges in two fetuses from the same dam at a single time point, 9 dpi (Table 1). Rare neutrophils infiltrating the meninges were immunoreactive for ZIKV in these two fetuses (Fig. 1B–C). Fixed sections of the same 2 fetuses exposed to ZIKV *in utero* were focally positive for ZIKV immunoreactivity (ir) in the fetal cortex (Fig. 1D–E, Table 1). The presence of viral antigen in the fetal cortex detected by ZIKV immunohistochemistry with peroxidase staining (Fig. 1D–E) was confirmed using immunofluorescent staining (Fig. 1G). Additional ZIKV IR was aslo observed in sections of fetal hippocampus and spinal cord in the same 2 fetuses (Fig. 1H–I). more consistent ZIKV IR was observed in the placenta (Fig. 1F, Table 1) as well as in the uterus (Table 1).

Additional ZIKV IR in serial coronal sections was only observed in the brains of the 2 fetuses that had previously stained positive for ZIKV IR, while all the other fetuses assayed were negative. Evaluation of serial sections of fetal brains did not identify any additional fetuses that were antigen-positive for ZIKV (data not shown).

### Growth and mortality rates of pups exposed in utero to ZIKV

**Figure 2:**
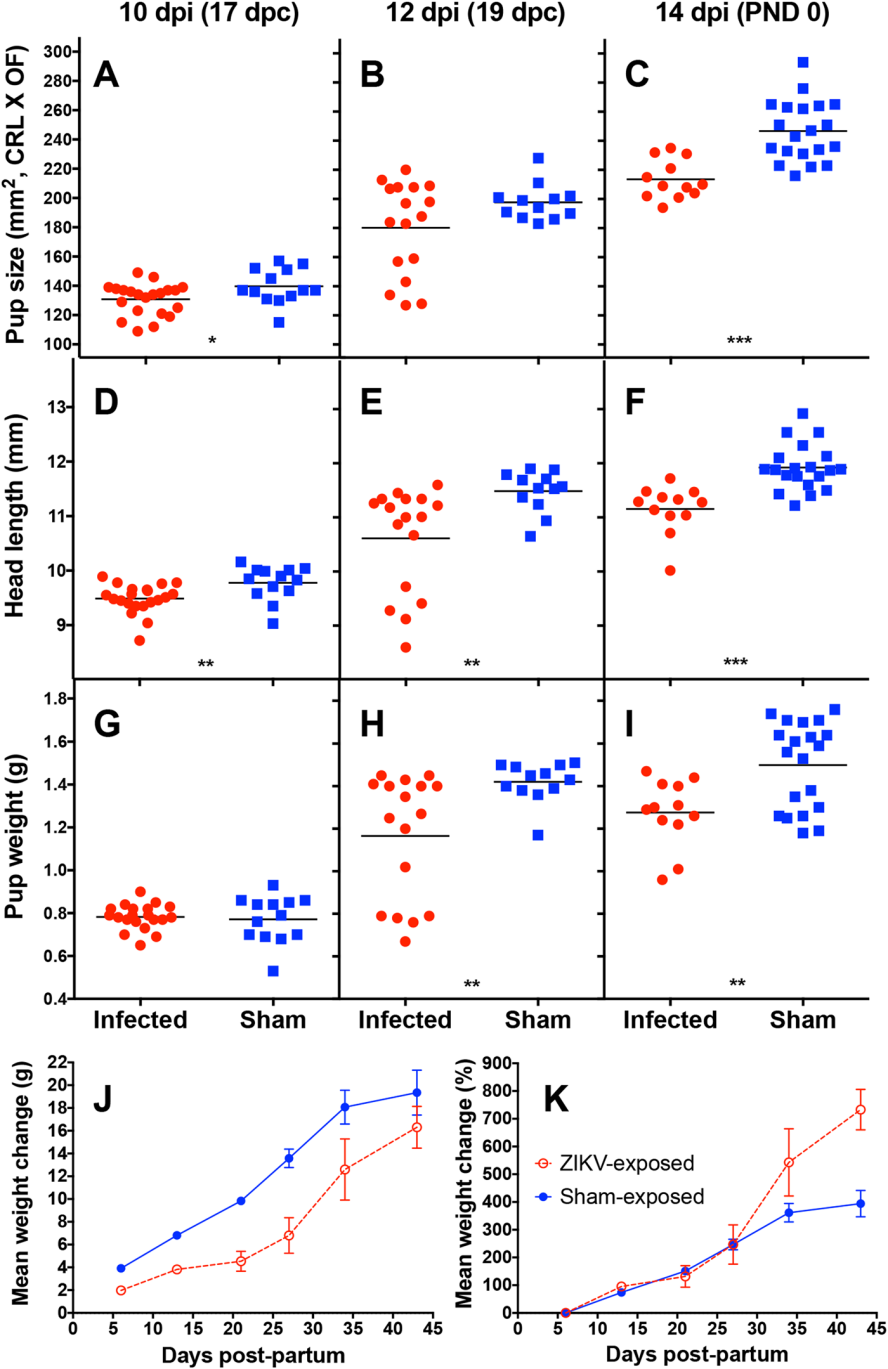
Growth parameters of ZIKV-exposed pups. Fetuses/neonates were measured 10, 12, or 14 dpi for (**A**–**C**) pup size, as indicated by crown-rump length X occipital-frontal diameter, (**D**–**F**) head length and (**G**–**I**) pup weight. Growth curves of pups from 7–45 days after birth are also shown and are expressed as (**J**) mean change in g weights or in (**K**) % weight.

Combined data from three independent studies provide insight into the growth rate of pups after congenital ZIKV exposure. Fetal and neonatal growth were impacted by congenital exposure to ZIKV, as indicated by measurement prior to and after birth including size (CRL × OF), head length and weight. Pups were smaller proportionately when compared with age-matched controls, which was significant (P ≤ 0.001) on post-natal day (PND) 0 (Fig. 2A–C). A similar trend in reduced head length of ZIKV-exposed pups was also observed, with significant differences observed between 10 dpi and parturition (Fig. 2D–F). These observations also corresponded with a reduced weight, which was significant (P < 0.01) just prior to or just after birth (Fig. 2G–I). Weight and size of neonates was occasionally observed to be reduced in ZIKV-exposed pups at various times after birth up to PND 16, but these parameters were quite variable. Consistent and significant differences in neonatal size between pups born to infected dams and controls were not observed in all experiments (data not shown). Mean percent growth rates were similar or more rapid for pups born to infected dams as compared with pups born to sham-infected controls, regardless of the smaller overall weight of pups born to infected dams (Fig. 2J–K).

**Figure 3:**
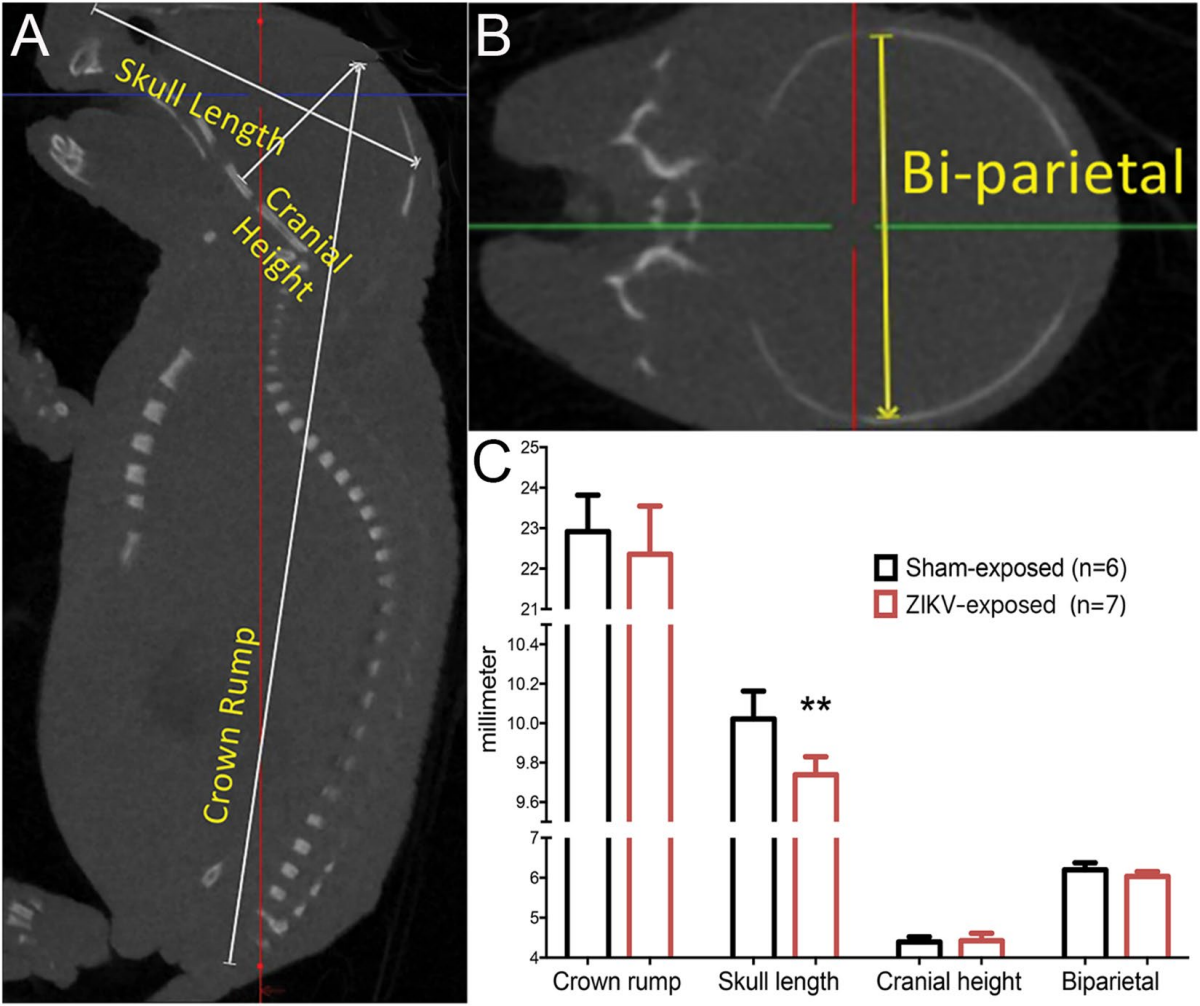
Micro-CT measurements of pups born to AG129 mouse dams injected with ZIKV at 7.5 dpc. For micro-CT analysis at PND 1, heads were fixed in paraformaldehyde and scanned for (**A**) skull length, cranial length, crown to rump length, and (**B**) biparietal lengths. (**C**) Averages of these measurements are graphically represented.

Skull length, cranial height, crown to rump length, and biparietal diameter were recorded and analyzed by micro-CT at PND1 (Fig. 3A,B). Skull lengths were statistically (P < 0.01) reduced in ZIKV-exposed pups on PND 1 as compared to neonates born to sham-infected controls, whereas the other measurements did not differ significantly between groups (Fig. 3C).

The mortality rate of pups born to ZIKV-infected dams was compiled from different studies and compared with the mortality rate of pups born to sham-infected control dams. Infected dams succumbed to viral illness within the first 7 days after giving birth, which necessitated the transfer of pups to surrogate dams. The AG129 mouse strain typically take good care of pups, and transfer of litters from an infected dam to a surrogate female typically resulted in successful fostering of many of these pups. A higher mortality rate, however, was observed in pups born to infected dams during the first week after birth (Table 2). It is difficult to distinguish if the observed mortality rate of 50% during this time was due to congenital infection of the pups, complications due to impaired intrauterine development, a lack of care from sick females or reduced care as a result of the transfer to surrogate females. Some instances of cannibalism by dams as well as surrogate females was also recorded and included in the total number of pups recorded as mortality <7 d column in Table 2. Pups born to infected dams had a 17% mortality rate after PND 7, as compared with a 3.8% mortality rate for pups born to sham-infected dams (Table 2). Ocular exudate and smaller body size was observed in a small number of neonates that later died or were euthanized. Mortality after PND 7 was generally not attributable to lack of maternal care, as by this time surrogate females had accepted the new pups and they were being fed and gaining weight (Fig. 2J–K). These results suggest that neonates congenitally exposed to ZIKV have a higher incidence of death as compared with age-matched controls.

**Table 2:** The effect of ZIKV infection on pregnancy outcome in AG129 mice.

ZIKV	#Dams^a^	#Pups	Stillborn (% of total)	Died < 7 d^b^ (% of survivors)	Died > 7 d (% of survivors)	Disease manifestations^c^
Infected	15	69	9 (13)	30 (50)	5 (17)	Small size, eye lacrimation (n = 4), reduced head length < P7
Sham-infected	15	64	5 (7.8)	7 (12)	2 (3.8)	None observed

^a^Data were taken from females included in 5 independent studies.

^b^Pups often died soon after birth due to neglect from female.

^c^Mouse pups were observed between P0 and P35 for disease.

### Assessment of hearing in congenitally exposed mice

**Figure 4:**
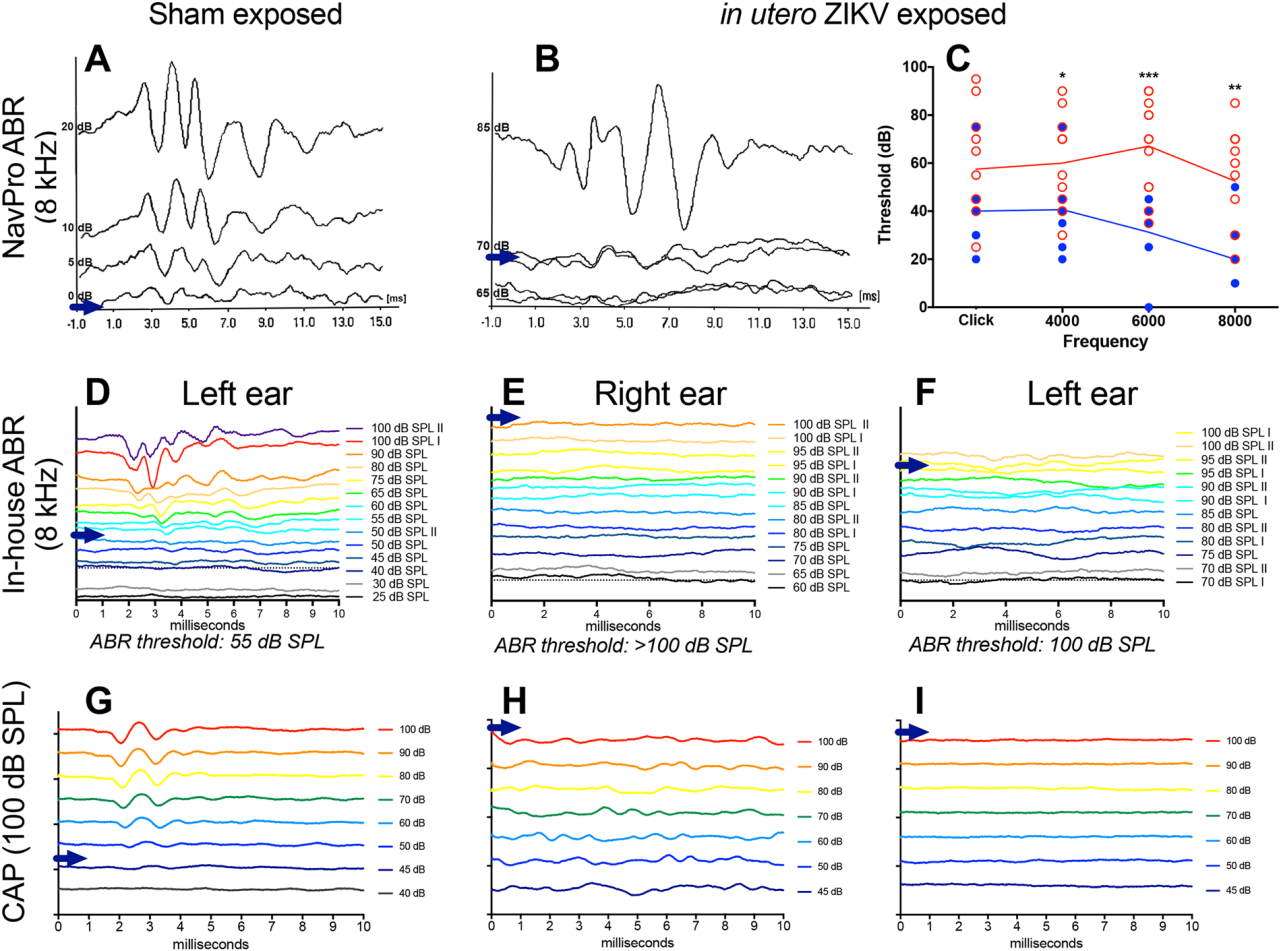
Hearing assessment of mice congenitally exposed to ZIKV. ABR hearing assessment was conducted on pups exposed *in utero* to ZIKV. Initial hearing assessment with a (**A**–**C**) NavPro system revealed (**A**) normal signal in mice born to sham-infected dams at low stimulus. (**B**) Representative data showing a threshold of 85 dB after exposure to an 8-kHz tone and (**C**) measurements of multiple ZIKV-exposed (red hollow circles) and sham-exposed (blue circles) mice at various tonal frequencies (***P < 0.001, **P < 0.01, *P < 0.05, as compared with sham-exposure). (**D**–**E**) An in-house developed ABR instrument was used to verify these data and representative data from a (**D**) sham-exposed control mouse and the (**E**) right and (**F**) left ear of a ZIKV-exposed mouse are shown. The same mice in (**D**–**F**) were measured using a (**G**–**I**) cochlear action potential (CAP) assay, which also demonstrated (**G**) normal hearing and (**H**–**I**) hearing deficits in these mice.

Weaned mice born to infected dams were evaluated for hearing loss between 8 and 16 weeks old. Hearing deficits, as determined by 2-standard deviations above average readings in normal age-matched controls, were detected using a NavPro ABR system in 25–66% of measurements of mice exposed *in utero* to ZIKV, depending on the frequency of the tone burst (Fig. 4A–C). A greater number of deficits were observed at higher frequencies (Fig. 4C). In several animals, deficits improved with subsequent measurements (data not shown). These animals were later re-tested using an in-house designed ABR instrument. No deficits were observed in these mice using click stimulus rather than tones of specific frequency, aside from a single animal, which had an ABR threshold of 80 dB in the left ear and 100 dB in the right ear (Fig. 4D–G). This animal was also found to have a hearing deficit during the initial phase of ABR assessment using the NavPro system.

In subsequent studies, mice congenitally exposed to ZIKV were evaluated for hearing deficits. Approximately 10% or less of mice had detectable hearing loss using click stimulus. The resolution of hearing deficits was also observed in these later studies. These results suggest that hearing loss in congenitally infected mice may be transient. However, a more long-term hearing loss was observed in a few individuals, suggesting chronic or permanent hearing loss may also occur.

Hearing deficits were confirmed in two mice using a cochlear action potential (CAP) assessment. This terminal procedure was performed on mice prior to collection of the cochlea for scanning electron microscopy. Mice that had a deficit in hearing obtained several weeks earlier also had deficiencies using the CAP assay (Fig. 4G–I). We have also used CAP to demonstrate hearing loss in an acutely-infected, adult AG129 mice (Suppl. Figure 1). Viral antigen was detected in cochlear sections (Suppl. Figure 1). Many of these animals were moribund, so it is difficult to say if the hearing loss was due to the late-stage disease, or if it was similar to hearing deficits seen in congenitally-exposed animals.

Confocal microscopy was used to evaluate and quantify the hair cells of the cochlea on 12 or 21 weeks after birth from four mice from two separate studies. These mice had exhibited profound hearing loss as detected by the NavPro system in the earlier study and the in-house constructed ABR instrument in the second study, which was confirmed using the CAP assay. No significant differences in the number of hair cells was observed in animals exposed congenitally to ZIKV as compared with age-matched controls born to sham-infected dams (Suppl. Figure 2). Hearing loss associated with ZIKV infection does not appear to involve damaged hair cells.

### Running wheel assay

The behavior of mice using a running wheel was used to evaluate motor function and circadian rhythm in ZIKV exposed mice and age-matched controls 16–17 weeks after birth. Mice exposed *in utero* to ZIKV had a similar activity rate as their sham-infected counterparts. No consistent irregularities in circadian rhythm were observed between groups (Suppl. Figure 3).

### Locomotor assay

We evaluated locomotion through analysis of walking patterns. Four out of 6 ZIKV-exposed mice analyzed for VPS score had no deficits (Suppl. Table 1). For the 2 ZIKV-exposed mice that had measurable deficits, the deficits were very mild (VPS scores of 1 or 2) and potentially below the level of reliable detection. Of the animals that were born to ZIKV-infected females and were assessed twice for VPS score, one pup that had deficit the first time it was measured, did not have an evident deficit when it was measured a second time. Additionally, sham-infected mice have also been shown to have mild deficits with scores of 1 and 2 in blinded VPS assessment (data not shown). No severe, consistent motor deficits were observed in ZIKV-exposed mice (Suppl. Table 2).

## Discussion

We have obtained live births from AG129 mice infected with ZIKV during pregnancy and have evaluated the offspring for size and growth comparisons as well as for functional deficits. Mice that were exposed to ZIKV *in utero* had hearing deficits as detected by ABR analysis. Not all animals had detectable deficits in hearing and appeared to resolve in the majority of animals. Of the 70 microcephalic infants born to mothers infected with ZIKV during pregnancy that were evaluated, only 6–7% were found with sensorineural hearing loss^30^. Hearing deficits were also found to occur after acute infection of adult patients, some of which resolved after the clearance of the virus^31^. The disease associated with ZIKV congenital infection appears to be different from that of other TORCH infections such as cytomegalovirus, which causes progressive hearing loss in experimentally infected neonatal mice^32^. Exposure to ZIKV *in utero* in AG129 mice can replicate hearing deficits observed in acutely infected or congenitally infected patients. Further studies are underway to better understand the mechanisms of hearing loss associated with ZIKV infection.

With a well-documented neurotropism, especially for fetal brains, we anticipated that cognitive or locomotor deficits associated with ZIKV congenital infection might be observed. Mice surviving several weeks after birth, however, did not have any statistically significant deficits detected using a running wheel analysis and mice exposed congenitally to ZIKV behaved similarly to age-matched controls. Analysis of offspring using a paresis scoring assay did not detect significant deficits, with observed abnormalities near the limit of detection for this assay. In an analysis of almost 1,300 pregnancies with known ZIKV exposure during pregnancy, only 5% of infants were found to have birth defects^7^, although this is much lower than previously reported rates of 46% rate of adverse outcome^33^. Microcephaly has been poorly characterized in mouse models, and while cortical thinning has been demonstrated^34^, it is difficult to know if severely afflicted fetuses and neonates survive long-term after birth to display symptoms of disease. Many fetuses are resorbed after ZIKV congenital infection, which has been attributed at least in part, to a functional interferon response^17^. While it is possible that an insufficient sample number was evaluated with each assay to reliably detect differences or that the assays were conducted too long after birth to detect abnormalities, it is also possible that mice that survive past the first week will progress normally.

One consistent observation of congenitally exposed offspring was smaller size at birth. Additionally, these mice also had significantly smaller skull length. A lower birth weight in newborns has also been commonly observed in women exposed to ZIKV during pregnancy^6^. Virus-induced pathology could contribute to fetal growth restriction, and infection of fetal brains could contribute to congenital defects, but this needs to be confirmed with perhaps more sensitive methods for *in situ* detection of virus in the brain. Recent studies have demonstrated potential mechanisms for the development of microcephaly in a mouse model^35^. Additionally, infection of the placenta can have significant consequences to the developing fetus. The host immune response may play a role in ZIKV congenital infection^17^. While not all features of congenital deformities as a result of ZIKV infection are replicated, the information gained from work in rodent models is indispensable to investigate potential mechanisms.

A time-course analysis of the effect of ZIKV congenital infection in AG129 mice, including the effect of intrauterine ZIKV exposure on viral infection of the fetus and placenta and the effect of virus exposure on growth at different stages of development, is provided. These data support findings of previous studies evaluating the effect of intrauterine infection after peripheral infection of rodent dams with ZIKV^25,26,36^. We provide additional details regarding the infection in the placenta and fetus and demonstrate a time-dependent increase in viral RNA in these tissues. Productive infection was observed when females were challenged with ZIKV on 7.5 dpc, which was similar to our experience with the distantly related flavivirus WNV in our previously published research^37,38^. Although these studies were conducted in an immunocompromised mouse strain, infection of AG129 mice models some features of natural congenital infection, including intrauterine growth restriction^39^ and can help to provide insights into prevention and treatment. Interestingly, the lack of IFN receptors in the AG129 mouse strain may actually allow ZIKV-exposed fetuses to survive to birth. Animals lacking IFN type I receptors infected on 5.5 dpc survived challenge, while heterozygous fetuses were typically resorbed, likely as a result of placental dysfunction due to IFN signaling^17^.

The virus replicated consistently to high titer in the placenta, although detection of viral RNA titer of the fetuses was less consistent and were often below the levels of detection. The most sensitive method of viral detection was QRT-PCR, although this does not necessarily represent viral replication. Some fetuses with high levels of viral RNA also had detectable ZIKV IR and infectious virus, while others had very little additional evidence of virus infection. Other investigators have also had difficulty detecting direct virus infection of the fetus using various methods, despite the presence of histopathologic lesions^40,41^. A number of scenarios may account for this, including the time of analysis or the limit of detection of the assays used. There is also a potential for genotypic and phenotypic changes of ZIKV after infection *in vivo* that make it more difficult to detect. This is further exemplified by the detection of ZIKV in fetuses by immune-electron microscopy after a lack of detection of virus infection by other methods^36^.

ZIKV IR was observed in neutrophils located in the meninges nearby an area ZIKV IR in the brain of a fetus from an infected dam. In a WNV study in mice^42^, investigators found that neutrophils support efficient replication of WNV, and are rapidly recruited to the site of WNV infection. Interestingly, viremia was diminished in mice depleted of neutrophils or in transgenic mice lacking an applicable neutrophilic chemokine receptor gene, which suggests that neutrophils may serve as a viral reservoir to disseminate the virus. However later in infection, the neutrophils may fill a traditional role in contributing to viral clearance. This experience with WNV infection may also apply in some aspects to ZIKV infection, which merits further investigations.

## Electronic supplementary material

Supplemental Materials

## References

[1] Calvet GA, Santos FB, Sequeira PC (2016). Zika virus infection: epidemiology, clinical manifestations and diagnosis. Current opinion in infectious diseases.

[2] Costa, F. *et al*. Emergence of Congenital Zika Syndrome: Viewpoint From the Front Lines. *Ann Intern Med*, 10.7326/M16-0332 (2016). 10.7326/M16-0332 PMC5444536 26914810

[3] Rasmussen SA, Jamieson DJ, Honein MA, Petersen LRZika (2016). Virus and Birth Defects–Reviewing the Evidence for Causality. N Engl J Med.

[4] Melo AS (2016). Congenital Zika Virus Infection: Beyond Neonatal Microcephaly. JAMA Neurol.

[5] Petersen LR, Jamieson DJ, Powers AM, Honein MA (2016). Zika Virus. N Engl J Med.

[6] Franca GV (2016). Congenital Zika virus syndrome in Brazil: a case series of the first 1501 livebirths with complete investigation. Lancet.

[7] Reynolds MR (2017). Vital Signs: Update on Zika Virus-Associated Birth Defects and Evaluation of All U.S. Infants with Congenital Zika Virus Exposure - U.S. Zika Pregnancy Registry, 2016. MMWR Morb Mortal Wkly Rep.

[8] Li, S. *et al*. Zika Virus Fatally Infects Wild Type Neonatal Mice and Replicates in Central Nervous System. *Viruses* **10**, 10.3390/v10010049 (2018). 10.3390/v10010049 PMC5795462 29361773

[9] Aliota MT (2016). Characterization of Lethal Zika Virus Infection in AG129 Mice. PLoS Negl Trop Dis.

[10] Bardina SV (2017). Enhancement of Zika virus pathogenesis by preexisting antiflavivirus immunity. Science.

[11] Dowall SD (2016). A Susceptible Mouse Model for Zika Virus Infection. PLoS Negl Trop Dis.

[12] Julander JG (2016). Efficacy of the broad-spectrum antiviral compound BCX4430 against Zika virus in cell culture and in a mouse model. Antiviral Res.

[13] Lazear HM (2016). A Mouse Model of Zika Virus Pathogenesis. Cell Host Microbe.

[14] Rossi, S. L. *et al*. Characterization of a Novel Murine Model to Study Zika Virus. *Am J Trop Med Hyg*, 10.4269/ajtmh.16-0111 (2016). 10.4269/ajtmh.16-0111 PMC4889758 27022155

[15] Jagger BW (2017). Gestational Stage and IFN-lambda Signaling Regulate ZIKV Infection In Utero. Cell Host Microbe.

[16] Miner JJ (2016). Zika Virus Infection during Pregnancy in Mice Causes Placental Damage and Fetal Demise. Cell.

[17] Yockey, L. J. *et al*. Type I interferons instigate fetal demise after Zika virus infection. *Sci Immunol* **3**, 10.1126/sciimmunol.aao1680 (2018). 10.1126/sciimmunol.aao1680 PMC6049088 29305462

[18] Julander JG, Siddharthan V (2017). Small-Animal Models of Zika Virus. J Infect Dis.

[19] Morrison, T. E. & Diamond, M. S. Animal Models of Zika Virus Infection, Pathogenesis, and Immunity. *J Virol* **91**, 10.1128/JVI.00009-17 (2017). 10.1128/JVI.00009-17 PMC5375682 28148798

[20] Fernandes NC (2017). Experimental Zika virus infection induces spinal cord injury and encephalitis in newborn Swiss mice. Exp Toxicol Pathol.

[21] Manangeeswaran M, Ireland DD, Verthelyi D (2016). Zika (PRVABC59) Infection Is Associated with T cell Infiltration and Neurodegeneration in CNS of Immunocompetent Neonatal C57Bl/6 Mice. PLoS Pathog.

[22] Yu, J. *et al*. Effective Suckling C57BL/6, Kunming, and BALB/c Mouse Models with Remarkable Neurological Manifestation for Zika Virus Infection. *Viruses* **9**, 10.3390/v9070165 (2017). 10.3390/v9070165 PMC5537657 28661429

[23] Cugola FR (2016). The Brazilian Zika virus strain causes birth defects in experimental models. Nature.

[24] Platt DJ, Miner JJ (2017). Consequences of congenital Zika virus infection. Current opinion in virology.

[25] Vermillion MS (2017). Intrauterine Zika virus infection of pregnant immunocompetent mice models transplacental transmission and adverse perinatal outcomes. Nat Commun.

[26] Siddharthan V (2017). Zika virus infection of adult and fetal STAT2 knock-out hamsters. Virology.

[27] van den Broek MF, Muller U, Huang S, Aguet M, Zinkernagel RM (1995). Antiviral defense in mice lacking both alpha/beta and gamma interferon receptors. J Virol.

[28] Basso DM (2006). Basso Mouse Scale for locomotion detects differences in recovery after spinal cord injury in five common mouse strains. J Neurotrauma.

[29] Hatzipetros, T. *et al*. A Quick Phenotypic Neurological Scoring System for Evaluating Disease Progression in the SOD1-G93A Mouse Model of ALS. *J Vis Exp*, 10.3791/53257 (2015). 10.3791/53257 PMC4692639 26485052

[30] Leal MC (2016). Hearing Loss in Infants with Microcephaly and Evidence of Congenital Zika Virus Infection - Brazil, November 2015–May 2016. MMWR Morb Mortal Wkly Rep.

[31] Vinhaes ES (2017). Transient Hearing Loss in Adults Associated With Zika Virus Infection. Clin Infect Dis.

[32] Wang Y (2013). A comparison of different murine models for cytomegalovirus-induced sensorineural hearing loss. Laryngoscope.

[33] Brasil P (2016). Zika Virus Infection in Pregnant Women in Rio de Janeiro. N Engl J Med.

[34] Li, C. *et al*. Zika Virus Disrupts Neural Progenitor Development and Leads to Microcephaly in Mice. *Cell Stem Cell*, 10.1016/j.stem.2016.04.017 (2016). 10.1016/j.stem.2016.10.017 27814481

[35] Gladwyn-Ng I (2018). Stress-induced unfolded protein response contributes to Zika virus-associated microcephaly. Nat Neurosci.

[36] Yockey LJ (2016). Vaginal Exposure to Zika Virus during Pregnancy Leads to Fetal Brain Infection. Cell.

[37] Julander JG (2005). Treatment of West Nile virus-infected mice with reactive immunoglobulin reduces fetal titers and increases dam survival. Antiviral Res.

[38] Julander JG (2006). West Nile virus infection of the placenta. Virology.

[39] Del Campo M (2017). The phenotypic spectrum of congenital Zika syndrome. Am J Med Genet A.

[40] Culjat, M. *et al*. Clinical and Imaging Findings in an Infant with Zika Embryopathy. *Clin Infect Dis*, 10.1093/cid/ciw324 (2016). 10.1093/cid/ciw324 PMC4996132 27193747

[41] Cugola, F. R. *et al*. The Brazilian Zika virus strain causes birth defects in experimental models. *Nature pre-publication release* (2016). 10.1038/nature18296 PMC4902174 27279226

[42] Bai F (2010). A paradoxical role for neutrophils in the pathogenesis of West Nile virus. J Infect Dis.

