# Supplementary material for: Consequences of in utero exposure to Zika virus in offspring of AG129 mice
Source: Sci Rep. 2018 Jun 20;8:9384. doi: 10.1038/s41598-018-27611-x (PMC6010449; doi:10.1038/s41598-018-27611-x)
Supplement: Supplementary file 1 — Supplemental Materials [file 41598_2018_27611_MOESM1_ESM.pdf]

Full title: Consequences of *in utero* exposure to Zika virus in offspring of AG129 mice

Short title: Effects of *in utero* Zika virus infection on offspring

Authors: Justin G. Julander[1]*, Venkatraman Siddharthan[1], Albert H. Park[2], Elizabeth Preston[3], Pranav Mathur[4], Michael Bertolio[1], Wang Hong[1], Katherine Zukor[1], Arnaud J. Van Wettere[5], Donal G. Sinex[6], John D. Morrey[1]

Affiliations and addresses:

[1] Institute for Antiviral Research, Animal, Dairy, and Veterinary Sciences Department, 5600 Old Main Hill, Utah State University, Logan, Utah, 84322-5600, USA

[2]Division of Otolaryngology- Head and Neck Surgery, University of Utah, 50 N Medical Dr., Salt Lake City, UT

[3]Audiology Division, Department of Communicative Disorders and Deaf Education, Utah State University, 2620 Old Main Hill, Logan, UT 84322-2620

[4]Department of Neurobiology and Anatomy, University of Utah, 1795 E South Campus Drive, Salt Lake City, UT 84112

[5]Utah Veterinary Diagnostics Laboratory, Utah State University, 950 E 1400 N, Logan, UT. 84341

[6]Department of Biology, Utah State University, 5305 Old Main Hill, Logan, UT. 84322-5305

*Corresponding author:

Justin G. Julander

justin.julander@usu.edu

435-797-7215

Supplemental Materials

Table 1. VPS scoring criteria

Score	Description	Signs
**0**	**normal**	**normal, weight-bearing, plantar stepping[a] with tail up during walking passes[b]**
**1**	**onset of symptoms**	**weight-bearing, plantar stepping with mild rotation**
	tail position:	may be down or not fully up
	miss-step:	foot rotated on take-off or landing
	weight bearing:	wobble is present indicating weakness
**2**	**mild paresis**	**mild miss-steps (but able to bear weight)**
	tail position:	down
	miss-step:	mild, toe curling/dragging on ground<br>foot slightly skids medially or laterally
	weight bearing:	a limp may be present indicating weakness
	joint movement:	may appear stiffer
**3**	**moderate paresis**	**moderate miss-steps (but able to bear weight)**
	miss-step:	obvious foot curling/dragging on ground<br>foot obviously skids medially or laterally
	weight bearing:	limb is obviously weak
	joint movement:	may appear stiffer
**4**	**severe paresis**	**severe miss-steps (not bearing much weight)**
	miss-step:	limb mostly drags behind, medially or laterally
	weight bearing:	not much, but limb still used to aid forward motion
	joint movement:	obviously decreased
**5**	**paralysis**	**no weight-bearing steps, slight joint movement**
	miss-step:	no stepping, limb only drags
	weight bearing:	none
	joint movement:	slight
**6**	**complete paralysis**	**no weight-bearing steps, no joint movement**
	joint movement:	none

[a] plantar stepping: Paw is placed flat on ground during stepping. It does not curl or skid to one side, and the toes/feet do not curl or drag at any point.

[b] walking pass: Animal moves 3 body lengths at a consistent speed and without turning.

Supplemental Figure 1. Hearing deficit and cochlear staining of acutely infected AG129 mice. (A) Significant (*$P<0.05$) increase in cochlear action potential (CAP) threshold in acutely infected AG129 mice as compared with controls. (B) Cochlea from infected AG129 mice stain positive for ZIKV. SGNs: spiral ganglion neurons, sl: spiral ligament, sv: stria vascularis, ihc: inner hair cell, ohc: outer hair cell. Scale bar: 100 µm.

Supplemental Figure 2. SEM of cochleas from in utero exposed mice. Loss of hair cells in the inner ear of animals born to ZikV infected dam. Percentage of missing outer hair cells in animals born to dams infected with Zika virus was similar to those born to sham infected dams (A). No loss of inner hair cells was observed. Representative images showing three rows of outer hair cells and one row of inner hair cells stained with phalloidin (green) in the organ of corti from animals born to zika infected dam (B) or sham infected dam (C). * indicates areas of missing hair cells. Scale bar - 20uM.

Supplemental Figure 3. Running wheel activity assay. Three ZIKV congenitally-exposed and 2 age-matched control mice were evaluated for activity and circadian rhythm using a running wheel that counted rotation in either direction. Data was collected every minute for 7 days. Activity patterns were consistent between groups, regardless of ZIKV exposure.

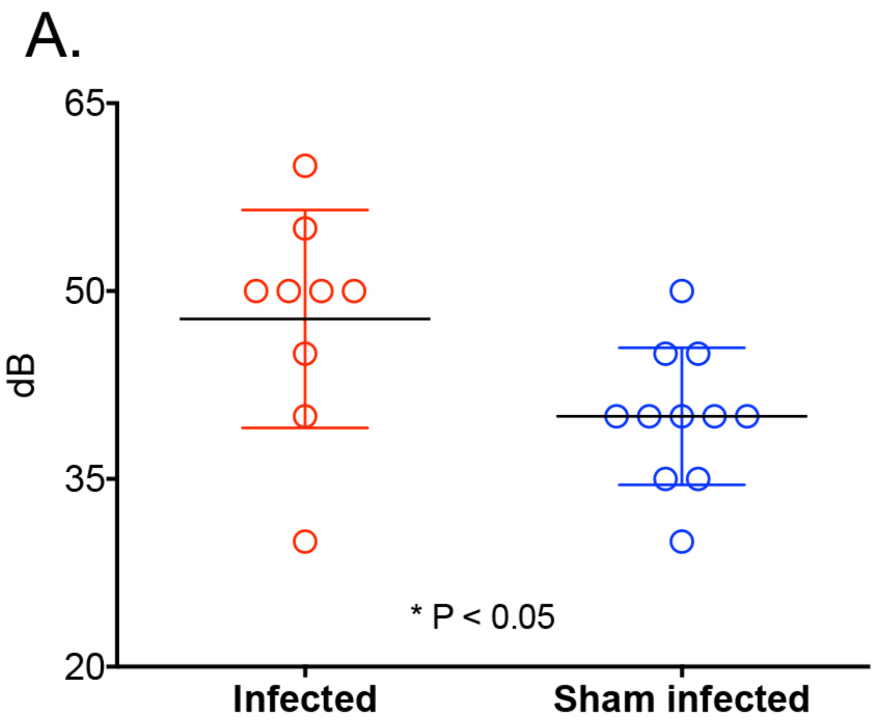

A.
65
50
35
20
dB
* P < 0.05
Infected
Sham infected

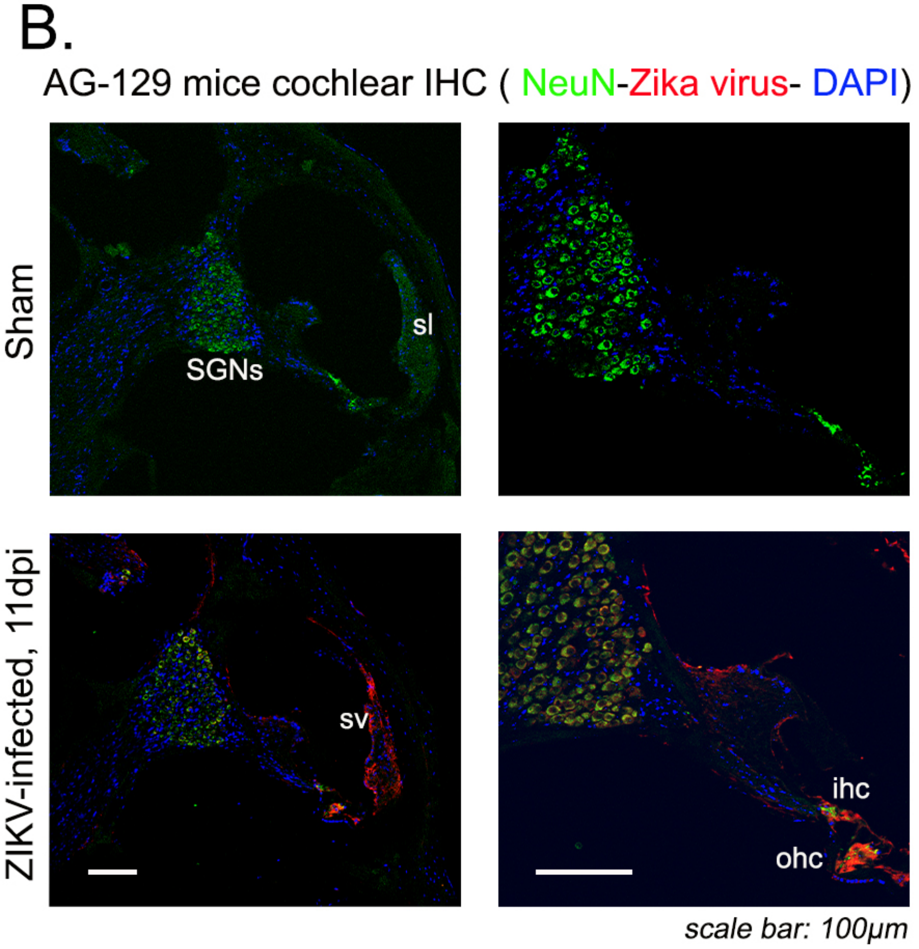

B.
AG-129 mice cochlear IHC (NeuN-Zika virus- DAPI)
Sham
ZIKV-infected, 11dpi
SGNs
sl
sv
ihc
ohc
scale bar: 100μm

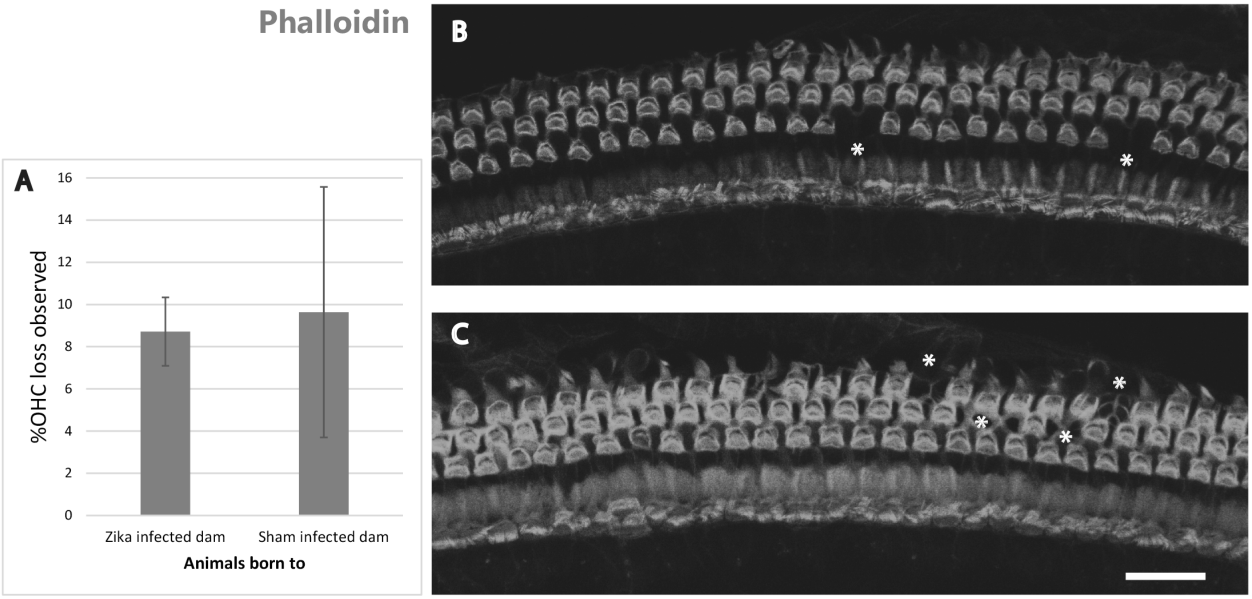
Phalloidin
A
%OHC loss observed
0
2
4
6
8
10
12
14
16
Zika infected dam
Sham infected dam
Animals born to
B
C

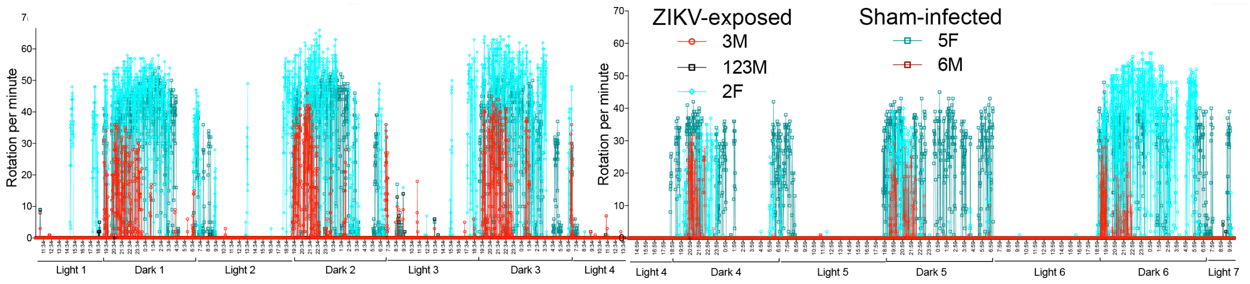

ZIKV-exposed
3M
123M
2F
Sham-infected
5F
6M
Rotation per minute
Rotation per minute
Light 1
Dark 1
Light 2
Dark 2
Light 3
Dark 3
Light 4
Light 4
Dark 4
Light 5
Dark 5
Light 6
Dark 6
Light 7
